# Distinct subtypes of endometriosis identified based on stromal-immune microenvironment and gene expression: implications for hormone therapy

**DOI:** 10.3389/fimmu.2023.1133672

**Published:** 2023-06-22

**Authors:** Yuning Wang, Yun Chen, Yinping Xiao, Jingyao Ruan, Qi Tian, Qi Cheng, Kaikai Chang, Xiaofang Yi

**Affiliations:** ^1^ Department of Gynecology, Hospital of Obstetrics and Gynecology, Fudan University, Shanghai, China; ^2^ Key Laboratory of Female Reproductive Endocrine Related Diseases, Hospital of Obstetrics and Gynecology, Fudan University, Shanghai, China

**Keywords:** endometriosis, heterogeneity, subtype, immune infiltration, fibroblast activation, unsupervised hierarchical clustering

## Abstract

**Background:**

Endometriosis (EMs) is a chronic inflammatory condition that is highly heterogeneous. Current clinical staging fails to accurately predict drug responses and prognosis. In this study, we aimed to reveal the heterogeneity of ectopic lesions and investigate the possible underlying mechanisms using transcriptomic data and clinical information.

**Methods:**

The EMs microarray dataset GSE141549 was obtained from the Gene Expression Omnibus database. Unsupervised hierarchical clustering was performed to identify EMs subtypes, which was followed by the functional enrichment analysis and estimation of immune infiltrates. Subtype-associated gene signatures were identified and further validated in other independent datasets, including GSE25628, E-MTAB-694, and GSE23339. Additionally, tissue microarrays (TMAs) were generated from premenopausal patients with EMs to investigate the potential clinical implications of the two identified subtypes.

**Results:**

The unsupervised clustering analysis revealed that ectopic EMs lesions can be classified into two distinct subtypes: stroma-enriched (S1) and immune-enriched (S2). The functional analysis revealed that S1 correlated with fibroblast activation and extracellular matrix remodeling in the ectopic milieu, whereas S2 was characterized by the upregulation of immune pathways and a higher positive correlation with the immunotherapy response. Moreover, we identified a subtype signature composed of FHL1 and SORBS1, and constructed a subtype diagnostic model. Based on the cohort data from the TMAs, we found that S2 was strongly associated with the failure of/intolerance to hormone therapy.

**Conclusions:**

This study identified two distinct subtypes that are varyingly associated with hormone resistance, stroma-immunity, and molecular features, thereby highlighting the importance of this stromal-immune heterogeneity in identifying EMs subtypes and providing novel insights into future personalized hormone-free therapy in EMs.

## 1 Introduction

Endometriosis (EMs) is a complex chronic condition that can cause dysmenorrhea, chronic pelvic pain, and infertility (1), and it affects approximately 10% of reproductive aged women worldwide (2). EMs is highly heterogeneous, and despite identical histology, comparable clinical characteristics, and uniform therapy, individual patient responses to hormone therapy can range from complete remission of symptoms to progression with treatment. Long-term disease management is a major clinical challenge, with first-line medical therapy (oral contraceptives and progestogens) being effective in only approximately 40% of patients who experience a major response, which therefore causes a delay in appropriate treatment (3, 4).

EMs has traditionally been classified based on anatomy (5). However, it remains a disease that lacks molecular subtypes to recapitulate the molecular profiles and present a basis for personalized treatment. With the development of next-generation sequencing, we now have the opportunity to further understand the heterogeneity and molecular mechanism of EMs. Several studies have explored the heterogeneity of EMs in the immune and stromal microenvironments (6, 7), and classification based on these features might provide new insights into its heterogeneous presentation (8).

In this study, we performed an unsupervised clustering analysis of EMs based on gene expression. We identified two subtypes of EMs that were associated with the clinical response to hormone therapy. The functional enrichment and cell infiltration estimation analyses revealed dysregulated pathways and cellular heterogeneity in the different subtypes. Furthermore, subtype-related genes were predicted and verified in a prospectively collected clinical cohort.

## 2 Materials and methods

### 2.1 Database collection

EMs transcriptomic microarray data were obtained from the NCBI’s Gene Expression Omnibus (GEO; https://ncbi.nlm.mih.gov/geo/), and data on the clinical features of patients examined in the microarray analyses were obtained from EMBL-EBI’s ArrayExpress (https://www.ebi.ac.uk/arrayexpress). The expression of genes of interest was analyzed in endometriotic tissue using the ENDOMET Turku Endometriosis Database (9). A total of 198 ectopic EMs lesion samples (including 6 biological replicates) from patients with EMs were analyzed from the GSE141549 dataset, which contains the expression matrix and clinical information of 198 EMs lesions. Additionally, three other independent transcriptomic profiles (GSE25628, E-MTAB-694, and GSE23339) were analyzed for further validation.

### 2.2 Batch effect removal

Expression values from both datasets were log2-transformed before cross-platform normalization. The ComBat function from the SVA package (in the R environment, version 3.6.2) (10) was used for the meta-analysis and data cleaning to remove batch effects. The principal component analysis (PCA) was used to evaluate whether the known batch effects were removed. Samples from the same dataset were considered to have no obvious batch effect if they did not cluster together.

### 2.3 Consensus clustering

Normalized expression values of 198 EMs lesions from the GSE141549 dataset were used to identify molecular subtypes by applying the consensus clustering method, which was implemented using the ConsensusClusterPlus package (in the R environment, version 1.58.0) (11). Consensus clustering was implemented with the following settings: maximum cluster number (maxK) = 10, number of repeats (reps) = 10,000, proportion of items to sample (pItem) = 0.8, proportion of features to sample (pFeature) = 1, cluster algorithm (clusterAlg) = “km” (K-means), and distance = “Euclidean”. The optimal cluster number was determined based on the consensus matrix and the cluster consensus score. The consensus score for k = 2 was larger than that of other clusters. Finally, the EMs lesions were clustered into two molecular subtypes: stroma-enriched subtype (S1) and immune subtype (S2).

### 2.4 Weighted gene coexpression network analysis

The biological function of each subgroup was investigated by identifying clusters of co-expressed characteristic genes using the WGCNA package (in the R environment, version 4.1.3) (12), which was also used to construct the co-expression network. The process also involved calculating the Pearson’s correlation matrices among all of the genes. Using the pickSoftThreshold function, 8 was determined to be the appropriate soft threshold to create the weighted adjacency matrix. Subsequently, hierarchical clustering and the dynamic tree cut function were used to assign genes to modules. The blockwiseModules function was applied using the following parameters: maxBlockSize = 30,000, minModuleSize = 30, mergeCutHeight = 0.25, reassignThreshold = 0.

Pearson’s correlation coefficients with P values estimated using the corPvalueStudent function were used to assess the correlations between molecular subtypes and module eigengenes.

The Kyoto Encyclopedia of Genes and Genomes (KEGG) pathway analysis and Gene Ontology (GO) analysis were performed using the clusterProfiler package (in the R environment, version 4.2.2) to explore the module-related pathways.

### 2.5 Analysis of cell type composition

Cell type composition scores were estimated for the S1 and S2 subtypes using the xCellAnalysis function from the xCell package (in the R environment, version 1.1.0) (13). Correlations between module eigengenes and computed cell type scores were visualized for all cell types that were scored in more than 25% of the samples, which allowed us to infer the representative cell type of each different module.

The infiltrating cells in lesions were also estimated using CIBERSORT (14), which yielded similar results to xCell.

### 2.6 Immune response prediction

The EaSIeR package (in the R environment, version 1.0.0) was used to predict the outcome of immune therapy for the different subtypes (15). EaSIeR provided the estimation of immune responses for each lesion based on RNA-seq data combined with prior knowledge, and lesions with a higher relative score had a stronger positive correlation with the immunotherapy response.

### 2.7 Gene set enrichment analysis and functional annotation

To explore the subtype-related biological processes, the GSEA (in GSEA software version 4.2.3) was conducted for each subtype (16). The KEGG pathway enrichment results were considered statistically significant based on the net enrichment score (NES), gene ratio, and P value. Gene sets with a |NES| > 1 and a NOM p < 0.05 were considered to be significantly enriched.

### 2.8 Protein–protein interaction network construction

The NetworkAnalyst (https://www.networkanalyst.ca/) online tool was used to construct and visualize the PPI network of gene models related to both subtypes (17). Genes with a connectivity degree of greater than 10 were identified as hub genes.

### 2.9 Identification of subtype-specific markers

Differentially expressed genes (DEGs) between the two different subtypes were identified using the limma package (in the R environment, version 3.50.1). Statistically significant DEGs were identified with the P value cutoff of <0.01 and a fold change of ≥2 or ≤−2.

### 2.10 Predictive signature identification

Subtype-related predictive genes were assessed in the GSE1141549 dataset using the glmnet package (in the R environment, version 4.1-4) based on the least absolute shrinkage and selection operator (LASSO) method and the randomForest package (in the R environment, version 4.1.3) based on Breiman’s random forest algorithm. The identified genes were used to build a linear regression model to examine the associations between the predictor genes and the molecular subtypes. The reproducibility was validated using the enhanced bootstrap method (100 bootstrap rounds). Subsequently, the model was tested on the three other datasets (GSE23339, GSE25628, and E-MTAB-694). C-statistics and Brier scores were calculated to assess the prediction performance. The C-index values were between 0.5 and 1.0, with 0.5 and 1.0 representing random opportunity and an excellent ability of the model to predict the subtype, respectively. The Brier score values were between 0 and 1, with 1 representing an entirely inaccurate forecast. A lower Brier score of a set of predictions indicated that the predictions were better calibrated, with the best possible Brier score of 0 representing total accuracy.

### 2.11 Patient cohorts and ethics

All tissue samples were obtained with informed consent from patients and approved by the ethics service under the research ethics committee numbers (kyy-2019-105). Premenopausal women diagnosed with EMs were included from the Obstetrics and Gynecology Hospital of Fudan University between January 2020 and September 2022. Samples were obtained from patients undergoing surgical resection of EMs lesions (conservative or radical surgery) and confirmed by pathological examination. All participants underwent hormone therapy before surgery. Detailed clinical information about previous EMs surgery and hormone therapy was collected for all recruited participants. Patients who underwent hormone therapy for less than 1 month were considered unexposed (18). The criteria for patients considered as failed/intolerant to hormone therapy were as follows: 1) patients who underwent hormone therapy for more than 1 month without any symptom relief and 2) patients who underwent hormone therapy for more than 1 month and who terminated treatment due to intolerable side effects.

Patients who met the following criteria were excluded: 1) patients with a history of any inflammatory condition, autoimmune disease, or malignant tumor; 2) patients who stopped treatment due to drug allergy or severe side effects; 3) patients who underwent hormone therapy for other conditions.

### 2.12 Construction of tissue microarrays

All tissues used to construct TMAs were evaluated by an experienced gynecological pathologist in advance of the presence of EMs lesions. When typical endometrial glands were found in the lesions, the pathological diagnosis of EMs was made. CD10 immunohistochemical staining of lesions was performed when the pathologists failed to find typical endometrial glands (e.g., due to heat injury). In total, 97 formalin-fixed, paraffin-embedded EMs lesions (22 intestinal lesions; 21 deep rectovaginal lesions; 11 other deep lesions, including ureteral or vaginal lesions; 36 ovarian lesions; and 7 peritoneal lesions) were used to construct the TMAs. The intestinal lesions were arrayed in two different paraffin blocks, whereas the deep rectovaginal lesions were arrayed in three different paraffin blocks. Ureteral lesions and vaginal lesions were arrayed in one paraffin block, ovarian lesions were arrayed in three different paraffin blocks, and peritoneal lesions were arrayed in one paraffin block. Tissue cylinders with a diameter of 5 mm were used to extract tissue from the targeted area confirmed by the pathologist of each donor tissue block, which were then deposited into recipient blocks. After the array blocks were constructed, they were cut into multiple 4-μm sections until all 97 tissue samples were represented on a single section. Each section was placed on a microscopic slide and histologically investigated after hematoxylin and eosin (H&E) staining to determine the adequacy of the arrayed tissues. These sections were separately placed on charged polylysine-coated slides for immunohistochemistry (IHC).

### 2.13 IHC analysis

The paraffin-coated microarray sections were placed on a heating block at 60°C for 2 hours and continuously washed with xylene to remove the paraffin. The slides were then rehydrated in varying concentrations of alcohol. Subsequently, 3% hydrogen peroxide was applied for 30 min at room temperature to block endogenous peroxidase activity. Then, the slides were incubated in the retrieval buffer (sodium citrate solution, pH = 6.0) and heated in the microwave to restore the antigen. The slides were pre-incubated with serum for 1 hour to reduce non-specific background. The slides were incubated overnight with primary antibodies diluted in phosphate-buffered saline (PBS) at 4°C. Four monoclonal antibodies were used to detecting PR (ab32085, 1:150), PI16 (ab127014, 1:500), FHL1 (ab133661, 1:100), and SORBS1 (ab224129, 1:200). The next day, the slides were washed three times with PBS (1×) for 5 min each. Subsequently, the slides were incubated with the secondary antibody (abs20040, 1:500) for 2 h at room temperature. The slides were developed in diaminobenzidine solution and stained with hematoxylin. Finally, images of the representative fields in each case were collected using the Stream Software, and expression was quantified using Image J software. The FHL1 and SORBS1 staining results were assessed as negative or positive by a pathologist who was unfamiliar with the clinical pathological data. Correlations between FHL1 or SORBS1 expression and the effects of hormone therapy were estimated using the chi-square test. Correlations between the expression of other genes and the effects of hormone therapy were estimated using the t-test, with or without Welch’s correction. All analyses were performed using GraphPad Prism v.9.0 software. P values of <0.05 were considered statistically significant.

## 3 Results

### 3.1 Data collection and preprocessing

**Figure 1:**
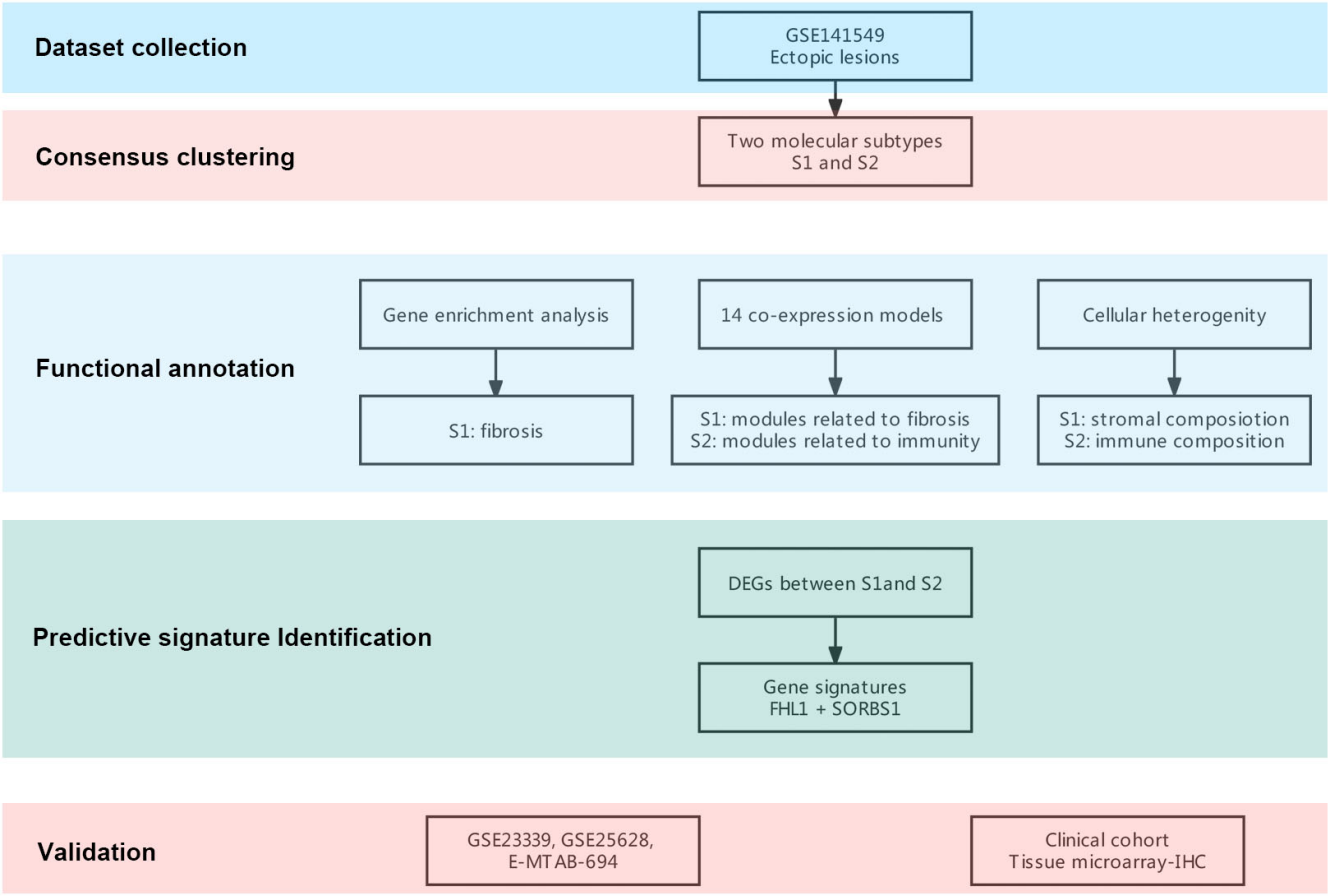
Flowchart of this research. A flowchart for the analysis procedure to identify potential molecular subtypes and critical genes.

**Figure 2:**
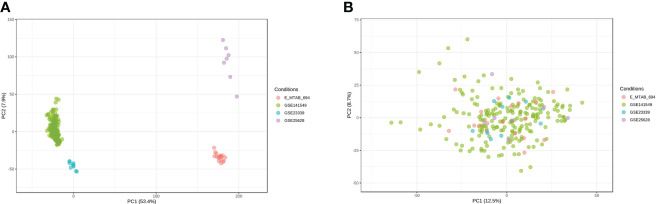
Removal of batch effects between the datasets. Principal component analysis (PCA) of the four datasets (GSE141549, GSE25628, E-MTAB-694, and GSE23339) before **(A)** and after **(B)** merging.

Microarray EMs transcriptomic data and matched clinical data were obtained from NCBI’s GEO (https://ncbi.nlm.mih.gov/geo/) and EMBL-EBI’s ArrayExpress (https://www.ebi.ac.uk/arrayexpress). In total, the data of 198 EMs samples (91 deep lesions, 79 peritoneal lesions, and 28 ovarian lesions) from the GSE1412549 dataset were used as the training dataset. The other datasets, including GSE25628 (7 deep lesions), E-MTAB-694 (18 peritoneal lesions), and GSE23339 (10 ovarian lesions), were used for further external validation. A detailed flowchart explaining the process is illustrated in **Figure 1**. To remove any batch effects caused by the data being from different platforms and different batches, the ComBat method was applied between the datasets. The PCA was also conducted on these datasets to establish the relationships among the four validation datasets. As shown in **Figure 2A**, samples from the four independent datasets formed different clusters before removing the batch effects; however, they clustered together after batch effect removal (**Figure 2B**). This clustering indicated that cross-platform normalization was successful in removing the batch effects.

### 3.2 Identification of ectopic lesion transcriptome-based subtypes for EMs patients

**Figure 3:**
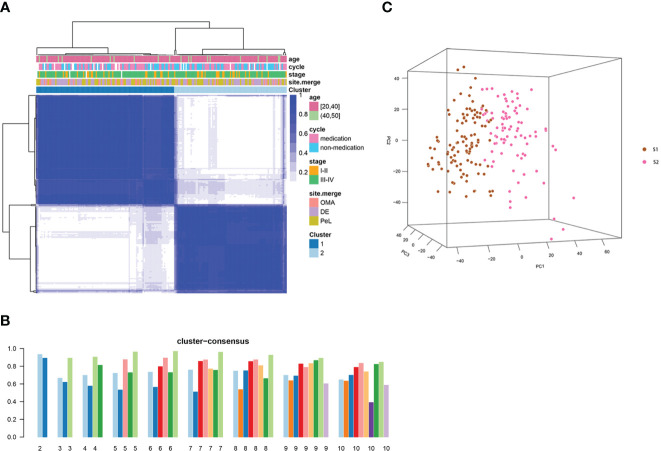
Consensus clustering analysis of the gene expression profiles for EMs cases. **(A)** The consensus matrix heatmap and consensus score were plotted when k = 2. The heatmap represents the consensus matrix with a cluster count of 2, which was determined by the minimal consensus scores of the subgroups (>0.8). **(B)** The bar plots represent the consensus scores of the subgroups with a cluster count ranging from 2 to 10. **(C)** The principal component analysis (PCA) supported the stratification when k = 2. “cycle”: cycle phase; “medication” refers to the inability to determine the cycle phase for patients on hormone medication; “non-medication” refers to the proliferative phase, secretory phase, or menstrual period; stage: rAFS stage; site.merge: location of lesions; cluster: molecular subtype.

To understand the heterogeneity of EMs and identify potential subtypes, the GEO dataset GSE141549 was downloaded along with clinical data (**Supplementary Table 1**) to screen EMs gene expression. EMs subtypes were identified from the high-dimensional dataset by applying the unsupervised clustering algorithm to classify the ectopic lesions based on the heterogeneity of the gene expression profiles. Normalized expression values of the 198 EMs lesions from GSE141549 were used to identify the different molecular subtypes. As shown in **Figure 3**, the EMs lesions were finally clustered into two molecular subtypes: S1 and S2. We observed that the S1 and S2 subtypes contained 109 and 88 lesions, respectively. The consensus matrix suggested a high degree of intra-group homogeneity and distinct heterogeneity between the two subtypes (**Figure 3A**). The cluster consensus value for each subgroup suggested that 2 was the optimal number of clusters (**Figure 3B**). The PCA analysis suggested that the two subtypes were separated at the transcriptional level (**Figure 3C**).

The detailed clinical characteristics of the 198 EMs patients along with clinical data from the GSE141549 dataset are shown in the additional file (**Supplementary Table 1**, **Supplementary Figure 1**). Given that the clinical characteristics might have an impact on the gene expression profile of lesions, the clinical characteristics of the two subtypes were investigated by examining the age, cycle, medication, revised American Fertility Society(rAFS) stage, and lesion locations of the EMs cases from the GSE141549 dataset. The proportion of lesion distribution in S1 was different from that in S2 (**Supplementary Table 1**). However, there were no significant differences between S1 and S2 with regard to age, menstrual cycle, and rAFS stage (**Supplementary Table 1**, **Supplementary Figure 2**). These results indicated that the transcriptome classification might represent certain intrinsic biological characteristics.

### 3.3 Identification of gene co-expression modules for each subtype and functional annotation

**Figure 4:**
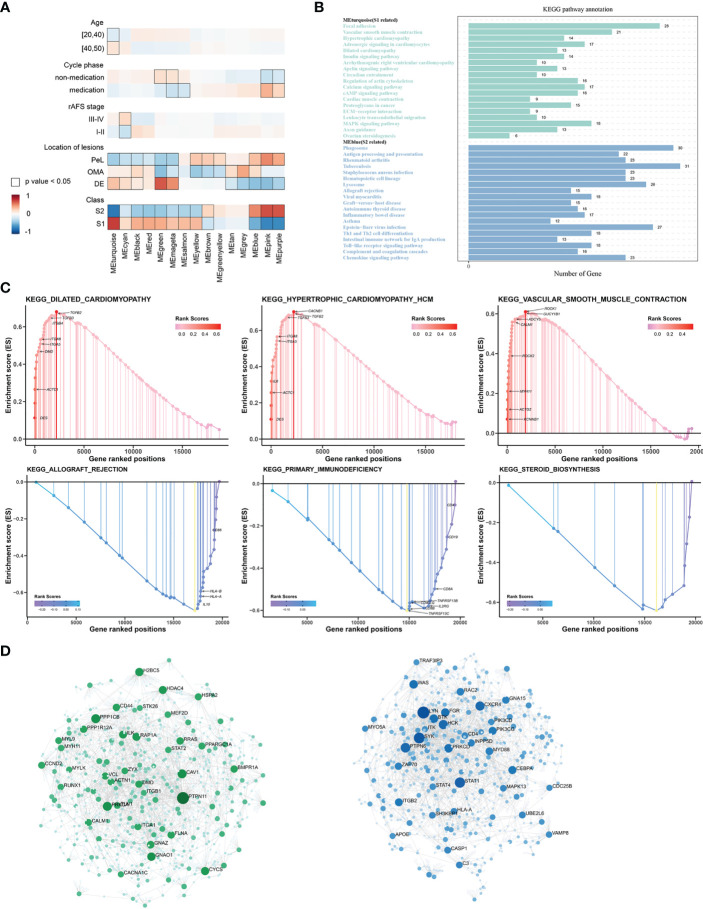
The underlying biological features differ between the two EMs subtypes. **(A)** Hierarchical cluster formation based on the soft threshold power (β = 8). Pearson’s correlation between module eigengenes and clinical features and cluster groups in lesion tissues within GSE141549, with bordered squares indicating significant correlations (P < 0.05, Student asymptotic P-value for correlation). Exact P values are given in **Supplementary Table 1**. Abbreviations: “cycle”: cycle phase; “medication” refers to the inability to determine the cycle phase for patients on hormone medication; “non-medication” refers to patients in phases of the menstrual cycle (not on hormone medication); “class”: molecular subtype. **(B)** Statistics of the KEGG pathway annotation of subtype 1 and subtype 2. The x-axis shows the number of genes; the y-axis corresponds to the KEGG pathway annotation. **(C)** The GSEA plots of representative gene sets in EMs subtypes. The green line indicates the enrichment profile. **(D)** PPI networks for gene co-expression modules associated with the molecular subgroups. The gene names of the top 30 nodes with the highest degree of connectivity are labeled. The top clusters from representative genes of subtype 1 (green color) and subtype 2 (blue color) are illustrated, and the related hub genes (dark green and dark blue) in each cluster that were obtained from degree values are shown.

The WGCNA was performed to identify clusters of co-expressed genes that were characteristic of the biological function of each subgroup. This process revealed 14 modules of highly co-expressed genes (**Supplementary Figure 3**). After calculating the correlations between each module and clinical traits, it was evident that several gene modules were strongly positively correlated with a subtype (MEturquoise for S1, MEblue for S2) (**Figure 4A**). The functional enrichment analysis based on the KEGG database indicated that genes in MEturquoise were mainly enriched in pathways related to fibrosis, whereas genes in Meblue were mainly enriched in pathways related to the immune response and inflammation (**Figure 4B**). The GO analysis showed similar results (**Supplementary Figure 4**).

The GSEA was performed for functional enrichment of S1 and S2 (**Supplementary Table 2**). The KEGG enrichment terms revealed that S1 was associated with fibrosis-related terms, including vascular smooth muscle contraction and dilated cardiomyopathy, whereas S2 was associated with immune response-related terms, including primary immunodeficiency and allograft rejection. Notably, steroid biosynthesis pathways were also enriched in S2 (**Figure 4C**).

### 3.4 Hub genes and immune-stroma profiles of the different established subtypes

Next, hub genes were identified from the representative genes of each subtype, and PPI networks were developed for gene co-expression modules associated with molecular subgroups; the gene names of the top 30 nodes with the highest degree of connectivity are labeled in **Figure 4D**. The hub genes identified for S1 included *PTPN11*, *PPKCA*, *CAV1*, *PPP1CB*, *PAP1A*, *CD44*, *CCND2*, and *PI16*, which might have the largest impact on fibroblast activation and integrin signal transduction in milieu. The hub genes identified for S2 included *LYN*, *STAT1*, *PTPN6*, *FGR*, *CXCR4*, *MYD88*, *CEBPA*, and *HCK*, which have an important function in regulating the innate and adaptive immune responses, responses to growth factors and cytokines, and migration of immune cells.

**Figure 5:**
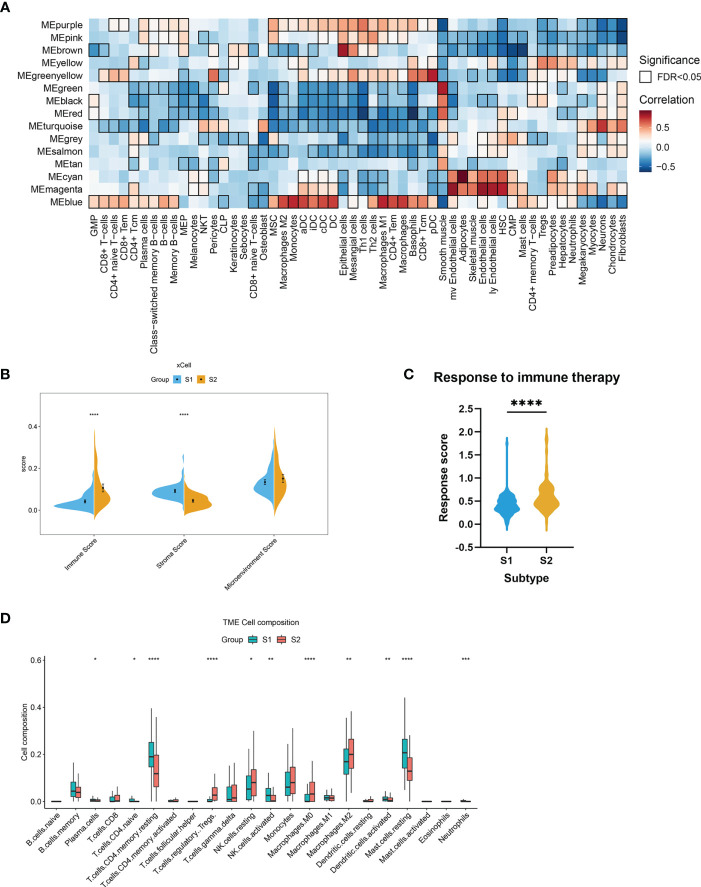
Stromal and immune cell composition alterations between the two EMs subtypes. **(A)** Heatmap of module eigengene–cell type correlations. The cell types were deconvoluted from whole-tissue expression data using xCell. The bordered squares indicate significant correlations (false-discovery rate [FDR] P < 0.05, asymptotic two-tailed P values estimated from Pearson’s correlation coefficients). **(B)** Comparisons of microenvironment, stromal, and immune scores between the two subtypes (S1 and S2). (***P < 0.001, ****P < 0.0001). **(C)** Immunotherapy response scores obtained by EaSIeR in the two subgroups of EMs patients. The differences in response scores between the two subtypes were identified using the Student’s t-test. **(D)** Boxplot of the proportion of 22 immune cell types in the different subgroups of lesions based on CIBERSORT. (*P < 0.05, **P < 0.01, ***P < 0.001, ****P < 0.0001).

We postulated that the observed gene co-expression patterns might also be indicative of cell type features associated with EMs. The differences in immune-related signatures between the EMs lesions of S1 and S2 were further explored. In this study, the xCell algorithm was used to analyze gene expression data (GSE141549) and calculate the module eigengene and immune-stroma scores of ectopic endometrial samples between S1 and S2. Strong positive correlations were observed between MEblue and obvious immune cell infiltration in S2, including that of macrophages and T lymphocytes (**Figure 5A**). The results revealed that the ectopic EMs lesions of S1 had significantly lower immune scores. In contrast, the stroma scores for ectopic EMs lesions of S1 were significantly higher than those of S2 (P < 0.0001). There was a significant difference between the microenvironment scores of the two subtypes (P > 0.05, **Figure 5B**). To further explore the association between the S2 subtype and the response to immunotherapy, we used the EaSIeR method to predict the immunotherapy effect of different lesions according to the gene expression profile. The results suggested that the immune signature score of the S2 subtype was higher than that of the S1 subtype, which might represent a better response to immunotherapy (**Figure 5C**). Similar results were obtained using the CIBERSORT algorithm (**Figure 5D**). Compared with the neutrophil enrichment in the S1 subtype, higher infiltrating M0 macrophage, M2 macrophage, Treg cell, and activated dendritic cell ratios were observed in the S2 subtype of the endometrial ectopic lesions, indicating the characteristic of immune tolerance.

### 3.5 Identification and validation of the DEGs between the EMs subtypes

**Figure 6:**
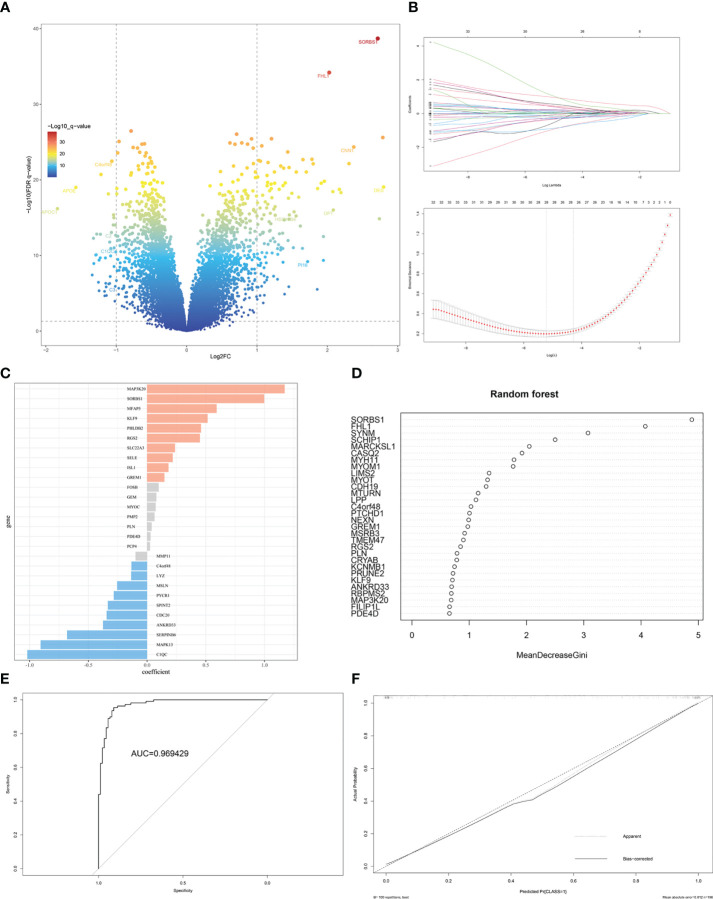
Identification of DEGs and gene-signature selection in EMs subtypes. **(A)** Volcano map of differentially expressed genes (DEGs) between subtypes S1 and S2. **(B)** The LASSO logistic regression algorithm was used to identify the most robust DEGs between different molecular subgroups, and an ensemble of key genes remained with individual coefficients. **(C)** The bar chart shows the variable weight of LASSO. **(D)** The variable weight of random forest. **(E)** The ROC curve for internal validation using the enhanced bootstrap method on the GSE141549 dataset. **(F)** Calibration curve for internal validation using the enhanced bootstrap method on the GSE141549 dataset. C index: 0.97, Brier score: 0.068.

The gene labels identifying the two subtypes were explored by performing differential analysis, which identified 159 DEGs. Of these, 120 DEGs were significantly upregulated in S1, whereas 39 DEGs were downregulated (**Figure 6A**, **Supplementary Table 3**). Subtype-related genes were identified using LASSO and the random forest algorithm based on the computed LASSO coefficient and the mean decrease in the Gini coefficient (**Figures 6B–D**). FHL1 and SORBS1 were selected to construct a predictive model using LASSO regression. These two genes were also differentially expressed between ectopic lesions and endometrium samples from patients (**Supplementary Table 4**). Internal validation computed by 100 rounds of the bootstrap method indicated excellent discrimination (area under the receiver operating characteristic curve [AUC]: 0.97) and calibration (0.068) (**Figures 6E, F**).

**Figure 7:**
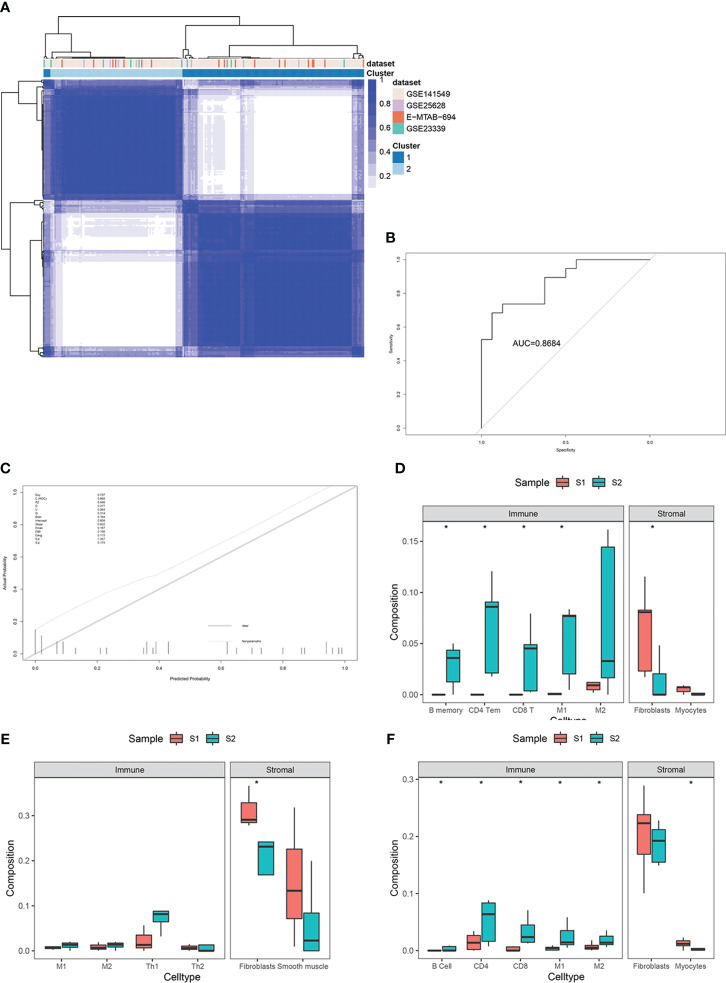
External validation in three independent datasets, including GSE25628, GSE23339, and E-MTAB-694. **(A)** Consensus clustering of lesion samples for the training dataset and the three validation datasets. **(B)** ROC curve for external validation using the enhanced bootstrap method in the validation datasets. **(C)** Calibration curve for external validation using the enhanced bootstrap method in the validation datasets. C index: 0.87, Brier score: 0.16. **(D–F)** Immune and stromal cell components of S1 and S2 in the validation datasets. *p value<0.05.

For external validation, three independent EMs datasets (GSE25628, GSE23339, E-MTAB-694) were analyzed to verify the predictive performance of this model. After removing batch effects among the datasets, consensus clustering of endometriotic lesion samples from the training dataset and the three independent validation datasets is shown in **Figure 7A**. The final results revealed that the AUC was 0.86 and the Brier score was 0.16 (**Figures 7B, C**).

Immune infiltration estimates for the validation datasets determined by xCell yielded similar results to those of the training set (**Figures 7D–F**).

### 3.6 Association between the EMs subtypes and clinical hormone therapy response

Hormone therapies are usually employed to treat EMs. However, our understanding of the molecular EMs subtypes and their associations with the clinical response to hormones remains incomplete. Furthermore, the significance of the EMs subtypes and their biomarkers requires validation across cohorts of well-annotated clinical samples and clinical features.

The unsupervised classification of the tissue transcriptome and gene signatures in EMs patients was validated by recruiting a validation cohort of 83 EMs patients. All patients participated in this study anonymously because of privacy and security concerns. The detailed baseline demographic information of the cohort is shown in **Table 1**. A total of 97 ectopic EMs samples were collected during surgery. On the basis of the above, we measured the protein expression of the subtype markers and functional molecules identified in **Figures 6A–D** using IHC and distinguished hormone-sensitive and hormone-resistant populations. In brief, with the help of IHC analysis of the TMAs, FHL1 and SOBRS1-positive and high PI16 expression patients were classified into the S1 subtype, while FHL1 and SORBS1-negative and low PI16 expression patients were classified into the S2 subtype.

**Table 1:** Clinical characteristics of the patients received hormone therapy before surgery.

	Overall (N=83)	Hormone therapy		P
		Responsive (n=59)	Failed/intolerant (n=24)	
**Age (years)^a^**	39.00 [36.00, 43.50]	39.00 [36.00, 43.50]	40.00 [35.50, 43.25]	0.988
**BMI (kg/m^2^)^a^**	21.78 [20.44, 23.53]	21.48 [20.44, 23.38]	22.91 [20.76, 26.95]	0.164
**Preoperative VAS** ≥5 **(%)**	57 (69.5)	39 (66.1)	18 (78.3)	0.419
**Previous surgery^b^ (%)**	45 (54.2)	31 (52.5)	14 (58.3)	0.813
Cycle phase (%)				0.408
Medicine	27 (32.5)	18 (30.5)	9 (37.5)	
Proliferative phase	37 (44.6)	29 (49.2)	8 (33.3)	
Secretory phase	19 (22.9)	12 (20.3)	7 (29.2)	
**Change in medicine^c^ (%)**	18 (21.7)	6 (10.2)	12 (50.0)	<0.001
**DNG (%)**	12 (14.5)	8 (13.6)	4 (16.7)	0.983
**IUD (%)**	20 (24.1)	5 (8.5)	15 (62.5)	<0.001
**OC (%)**	22 (26.5)	13 (22.0)	9 (37.5)	0.241
**GnRHa (%)**	59 (71.1)	42 (71.2)	17 (70.8)	1
The length of GnRHa prescription (%)				0.565
<3 months	35(42.1)	25(42.4)	10(41.7)	
3-6 months	42 (50.6)	31 (52.5)	11 (45.8)	
>6 months	6 (7.2)	3 (5.1)	3 (12.5)	
**CA125^a^ (mIU/ml)**	35.73 [23.50, 73.11]	34.58 [23.45, 68.04]	37.42 [24.86, 77.98]	0.682
**AMH^a^ (ng/ml)**	1.27 [0.39, 2.49]	1.31 [0.52, 2.55]	0.88 [0.23, 2.11]	0.245
**Combined adenomyosis^d^(%)**	61 (73.5)	44 (74.6)	17 (70.8)	0.939
**Combined DE^e^(%)**	69 (83.1)	49 (83.1)	20 (83.3)	1
Surgical complexity^f^(%)				0.642
A	3 (3.6)	3 (5.1)	0 (0.0)	
B	30 (36.1)	21 (35.6)	9 (37.5)	
C	37 (44.6)	25 (42.4)	12 (50.0)	
D	13 (15.7)	10 (16.9)	3 (12.5)	

^a^median [IQR]; ^b^patients with a medical history of abdominal surgery; ^c^patients who switched hormone therapies before surgery; ^d^adenomyosis detected during surgery; ^e^deep EMs detected during surgery; ^f^AAGL 2021 Endometriosis Classification (19). “Medicine” refers to patients who underwent hormonal therapy within 3 months before surgery and therefore did not have normal menstrual cycles. DNG, dienogest; LNG-IUD, levonorgestrel-releasing intrauterine system; OC, oral contraceptive; GnRHa, gonadotropin-releasing hormone analog.

**Figure 8:**
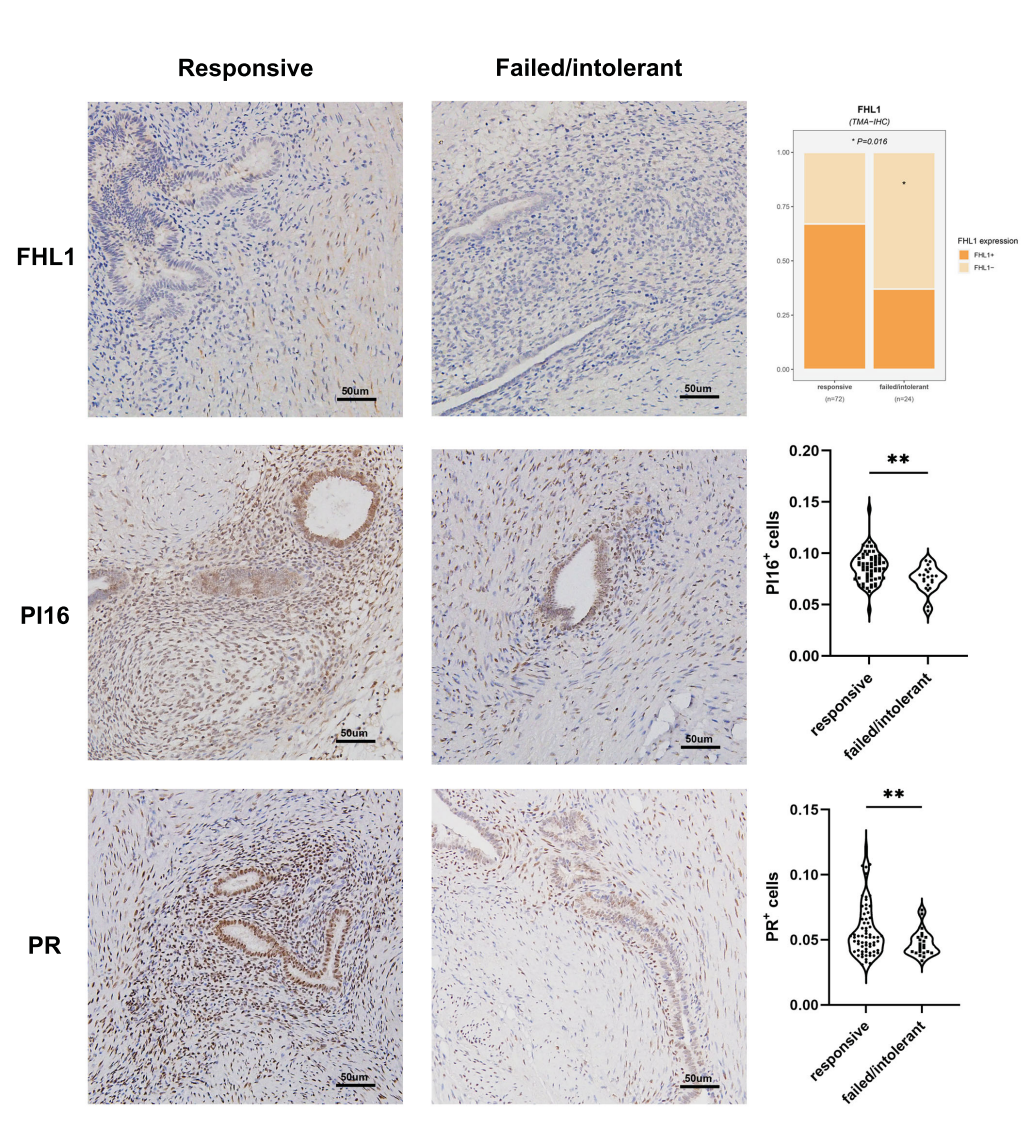
Correlation between EMs subtypes, molecular pathology characteristics, and validation. FHL1 expression in ectopic lesions from patients with hormone resistance or a clinical response (top). Progesterone receptor (PR; middle) and universal fibroblast marker PI16 (bottom) expression was detected in the tissue microarrays using IHC data from patients in the clinical cohort. *p value<0.05; **p value<0.01.

The subtype-specific gene signatures were identified in all patients. The associations between established EMs subtypes and clinical characteristics are reported in **Table 1**. Overall, the subtypes were significantly associated with the lesion locations (P < 0.001). Next, the correlation between FHL1 positivity and the response to hormone therapy was examined for ectopic EMs lesions. A higher number of hormone-resistant EMs patients were found in FHL1-positive subgroup (S2) (**Figure 8**, top), which is consistent with a previous report showing that chronic inflammation might induce a progesterone-resistant state. However, we did not observe any correlation between SORBS1 expression and the response to hormone therapy. As recent research has reported in human tissues, a new universal fibroblast transcriptional subtype was identified across tissues. The PI16-positive fibroblast serves as a reservoir that can yield specialized fibroblasts across a broad range of steady-state tissues and activated fibroblasts under pathological conditions. In this study, IHC was applied to verify the expression of the universal fibroblast marker PI16, the same as PR, which was significantly decreased in the ectopic EMs tissues from resistant patients (**Figures 8**, middle and bottom). Overall, the highest numbers of endometrial stromal cells and fibroblasts were observed in the FHL1-positive subtype, indicating a subtype-specific response, which was likely involved in the distinct biological behavior of the different EMs subtypes.

## 4 Discussion

EMs is a highly heterogeneous disease with distinct clinical manifestations and a complex pathophysiology (1, 20). More than 50% of women with chronic pelvic pain are estimated to suffer from EMs; however, correlations between clinical symptoms and the location, hormone therapy, and clinical classification of EMs lesions remain poorly studied, with some women being asymptomatic or progesterone-resistant. Most research considers EMs as a single disease phenotype, potentially obscuring informative subtype-specific associations that can identify risk factors, biomarkers, and treatment responses (21–25). In addition, traditional histological classification cannot guide EMs treatment, with the hormonal responses being ineffective in approximately 2%–30% of cases (4, 25–27). Therefore, accurately identifying the molecular subtypes of EMs is essential to make a lesion-based therapeutic decision, thereby possibly maximizing patient adherence and treatment results.

In this study, substantial heterogeneity of the ectopic tissue transcriptome in EMs patients was revealed (**Supplementary Table 1**), along with the identification of two distinct subtypes based on the ectopic tissue gene expression profiles (**Figure 3**). To understand and identify the biological processes and pathways involved in the different subtypes, KEGG pathway annotation statistical analyses were conducted (**Figure 4**). The two EMs subtypes presented with distinct molecular pathways, stroma compositions, immune cell infiltration patterns, and immune response patterns during ectopic lesion formation. Distinct fibrosis and immune infiltration patterns were observed between the two EMs subtypes. These distinct patterns were observable across the entire endometrial host response process, including the activation of stromal cells and fibroblasts, the infiltration and response of immune cells, and the production of extracellular matrix.

Consistent with previous research reports (23, 28), signaling pathways in fibrosis-myofibrosis were evidently enriched in S1 and were involved in the process of EMs-related fibrosis and adhesion. Moreover, strong enrichment in primary immunodeficiency and allograft rejection were observed in S2. This may imply that the modulation of signaling in innate and adaptive immunity mainly mediates the inflammation and progression of S2.

EMs heterogeneity was also confirmed by the differential analysis of immune cell proportions. The proportion of each cell type in ectopic tissue, especially stroma and immune cells, was found to shape significant differences between the two subtypes (**Figure 4**). Macrophages are crucially involved in lesion establishment and maintenance by driving chronic inflammation and tissue remodeling. Similarly, higher proportions of M2 macrophages and Treg cells were observed in lesions of the S2 subtype (**Figure 5**), indicating immunoregulatory/immunosuppressive properties. Based on our previous research on the role of Treg cells (29) and Th17 cells in EMs (30), this bioinformatic study further validated that the crosstalk between ectopic endometrial cells and immune cells creates an atmosphere of endometriotic immunotolerance in the ectopic milieu, which contributes to EMs progression.

To identify more stable and reliable molecular markers, 159 DEGs were identified, including 120 upregulated and 39 downregulated genes. DEGs between the two subtypes were closely associated with cell adhesion, cell junction, and fibroblast-myofibroblast activation. Two-gene signatures that discriminated these two EMs subtypes were selected and validated. A two-gene signature (FHL1 and SORBS1) was developed through stability selection and LASSO logistic regression (**Figure 6**). FHL1, a member of the four-and-a-half-LIM-only protein family, might be involved in muscle development or hypertrophy. Another study showed that FHL1 promotes blastocyst-epithelial adhesion (31). Therefore, we speculated that FHL1 might mediate the excessive adhesion of endometrial epithelial cells to the ectopic milieu, which is the opposite role. This two-gene signature was further validated to be stable in discriminating between these two subtypes, even in the other three independent datasets (**Figure 7**).

To date, several causes of progesterone resistance in patients with EMs have been postulated, including congenital genetic causes (PR gene polymorphisms and epigenetic modifications) and secondary progesterone-resistant states induced by chronic inflammation (26). Considerable evidence reveals a link between progesterone resistance and chronic inflammatory states among patients with EMs. Repetitive retrograde endometrial shedding is known to beget chronic peritoneal inflammation, which further exacerbates progesterone resistance. This could be partly caused by higher expression of hormone receptors on the stroma-enriched areas and glands, as well as the alteration of the PRs through chronic inflammation. Cytokines directly decrease PR expression, possibly through epigenetic modification (32) or disruption of receptor function through alterations in steroid receptor chaperone proteins (33). In addition to the hormone-resistant properties of the lesions, undiscovered deep-hidden microlesions, pelvic adhesion, concurrent chronic pelvic inflammation or uterine adenomyosis may also easily lead to the poor response to hormone therapy. Inflammation and adverse microenvironmental factors may also be implicated in hormone unresponsiveness *via* alterations in the progesterone response (26, 34). Despite the development of highly selective progesterone, congenital or acquired hormone unresponsiveness remains a challenge when treating partial-subtype EMs. We believe that in the near future, nonsurgical therapy for EMs requires innovation and expansion into new areas. The first step should involve obtaining a deeper understanding of the disease’s core features and diverse phenotypes and idiosyncrasies.

In this work, FHL1-positive dominant fibroblasts represented the stroma-enriched subtype S1, which was obviously associated with a robust hormone response and transcriptome. In contrast, subtype S2 with immune infiltration and immune tolerance signatures was prone to hormone tolerance (**Figure 8**). This result might have been partly caused by the higher expression of hormone receptors on the stroma and alteration of PRs through chronic inflammation.

New research has shown that PI16-positive fibroblasts represent a universal subtype that is responsible for extracellular matrix secretion and potentially serving as a resource cell that can develop into specialized fibroblasts (35). Given the significant differences in the stroma-immune cell compositions between the two subtypes, the abundance of PI16-positive fibroblasts in the subtypes were tested, and their association with progestogen resistance was explored. Using IHC assays through TMAs, this study identified a two-gene signature that discriminated these two subtypes when combined with the universal fibroblast marker PI16, which were able to predict the clinical hormone response of EMs patients. However, we emphasize that the assessment of the significance for hormone therapy might have been limited by the sample size, and further research is warranted in the future.

It is also worth mentioning that our analysis was based on microarray data, and the tissues surrounding the lesions might have affected the sequencing results. We analyzed the anatomical locations of the lesions in the two subtypes as a supplement, and we found that most of the deep lesions were classified as S1, while ovarian lesions were classified as S2. Notably, there was no obvious relationship between peritoneal lesions and molecular subtypes, as these lesions were equally distributed in the subtypes (**Supplementary Figure 5A**). Moreover, during the validation of the public database (GSE23339, GSE25628, and E-MTAB-694), we included datasets containing sequencing data of EMs lesions from three anatomical sites, including deep lesions (GSE25628), ovaries (GSE23339), and peritoneum (E-MTAB-694). We found no obvious correspondence between the lesion location and the molecular subtype (**Supplementary Figure 5B**). From another perspective, current classification was poorly correlated with symptom severity and failed to provide information concerning the prognosis or treatment response. Likewise, the classifications based on the anatomical location did not include any information about the molecular features or microenvironment of these lesions, nor did they fully recognize the etiology of the disease. We have come to realize that EMs has high potential for molecular heterogeneity, including mutations, gene expression profiles, and intra-lesion spatial heterogeneity (such as that in a large endometrioma or deep nodules) (36). We believe the heterogeneity of the lesion itself, rather than the anatomical location, is more likely to drive the diversity and heterogeneity of EMs and underpin the different molecular subtypes.

Another limitation is that intra-lesion heterogeneity in endometriosis has been reported by researchers, although it has not been studied extensively (36–38). In the dataset GSE141549 used in this study, 198 lesions contained 192 independent lesion tissues and 6 biological replicates. We found differences in gene expression profiles even between tissues derived from the same lesion. We believe these replicates derived from the different sampling sites within the same lesion, and such difference may partly reflect the intra-lesion heterogeneity. However, large-scale studies, as well as single-cell sequencing and spatial transcriptomics are needed for the further understanding of intra-lesion heterogeneity in endometriosis.

In summary, the integrated bioinformatics analysis identified candidate DEGs and pathways in EMs that enhance our understanding of the underlying molecular events of EMs. These candidate genes and pathways could be therapeutic targets for EMs. The classification of EMs into two subtypes further improves our understanding of the underlying pathogenesis of EMs and provides new insights for future studies.

## Data availability statement

The datasets presented in this study can be found in online repositories. The names of the repository/repositories and accession number(s) can be found below: GSE141549,GSE25628, and GSE23339 (GEO) and E-MTAB-694 (EMBL-EBI).

## Ethics statement

The studies involving human participants were reviewed and approved by the institutional ethics committee of Obstetrics and Gynecology Hospital, Fudan University. The patients/participants provided their written informed consent to participate in this study.

## Author contributions

YW and KC designed this study and took responsibility for the integrity of the data and the accuracy of the data analysis. YW performed the bio-informatics analyses and drafted the manuscript. YX and YW assisted the IHC of TMAs. YC assisted the recruitment,enrollment, and follow-up of clinical patients. QT, JR and QC assisted the collection of clinical information of patients, XY and KC revised the manuscript. All authors contributed to the article and approved the submitted version.
