# Supplementary material for: Distinct subtypes of endometriosis identified based on stromal-immune microenvironment and gene expression: implications for hormone therapy
Source: Front Immunol. 2023 Jun 22;14:1133672. doi: 10.3389/fimmu.2023.1133672 (PMC10324653; doi:10.3389/fimmu.2023.1133672)
Supplement: Supplementary file 1 [file DataSheet_1.docx]

Supplementary Material

Distinct Subtypes of Endometriosis Identified Based on Stromal-Immune Microenvironment and Gene Expression: Implications for Hormone Therapy

Yuning Wang1†, Kaikai Chang1,2†, Yinping Xiao1, Yun Chen1, Jingyao Ruan1, Qi Tian1, Qi Cheng1, Xiaofang Yi1,2*

*** Correspondence:** Xiaofang Yi: yix@fudan.edu.cn

# Supplementary Figures and Tables

## Supplementary Figures


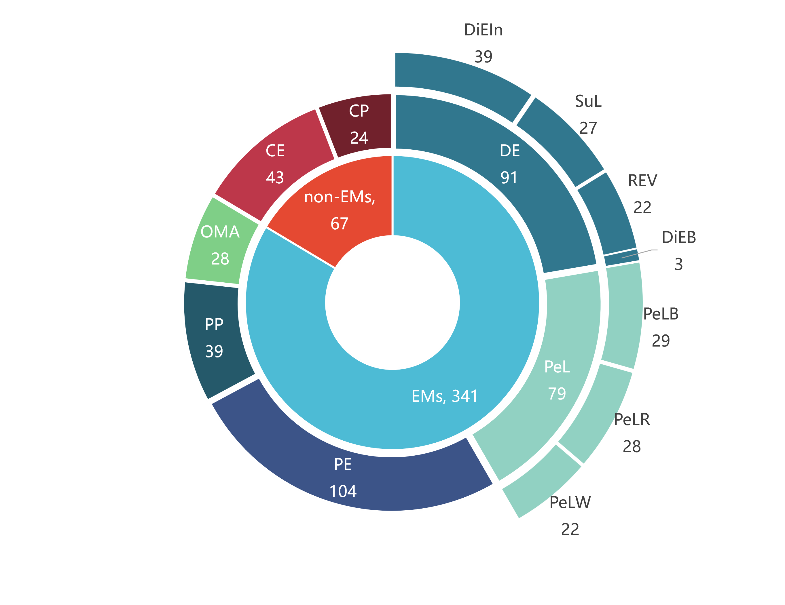


Supplementary Figure 1. Sample tissues of patients with endometriosis and healthy controls used in GSE141549 database. EMs = endometriosis; OMA = ovarian endometrioma; PeL = peritoneal endometriosis lesions; PeLR = red peritoneal endometriotic lesion; PeLB = black peritoneal endometriotic lesion; PeLW = white peritoneal endometriotic lesion; DE = deep endometriosis; REV = deep rectovaginal lesion; SuL = sacrouterine ligament lesion; DiEIn = intestinal endometriotic lesions; DiEB = deep endometriotic lesions in the bladder; PE = endometrium samples from patients; CE = endometrium samples from healthy controls; PP = peritoneum samples from patients; CP = peritoneum samples from healthy controls


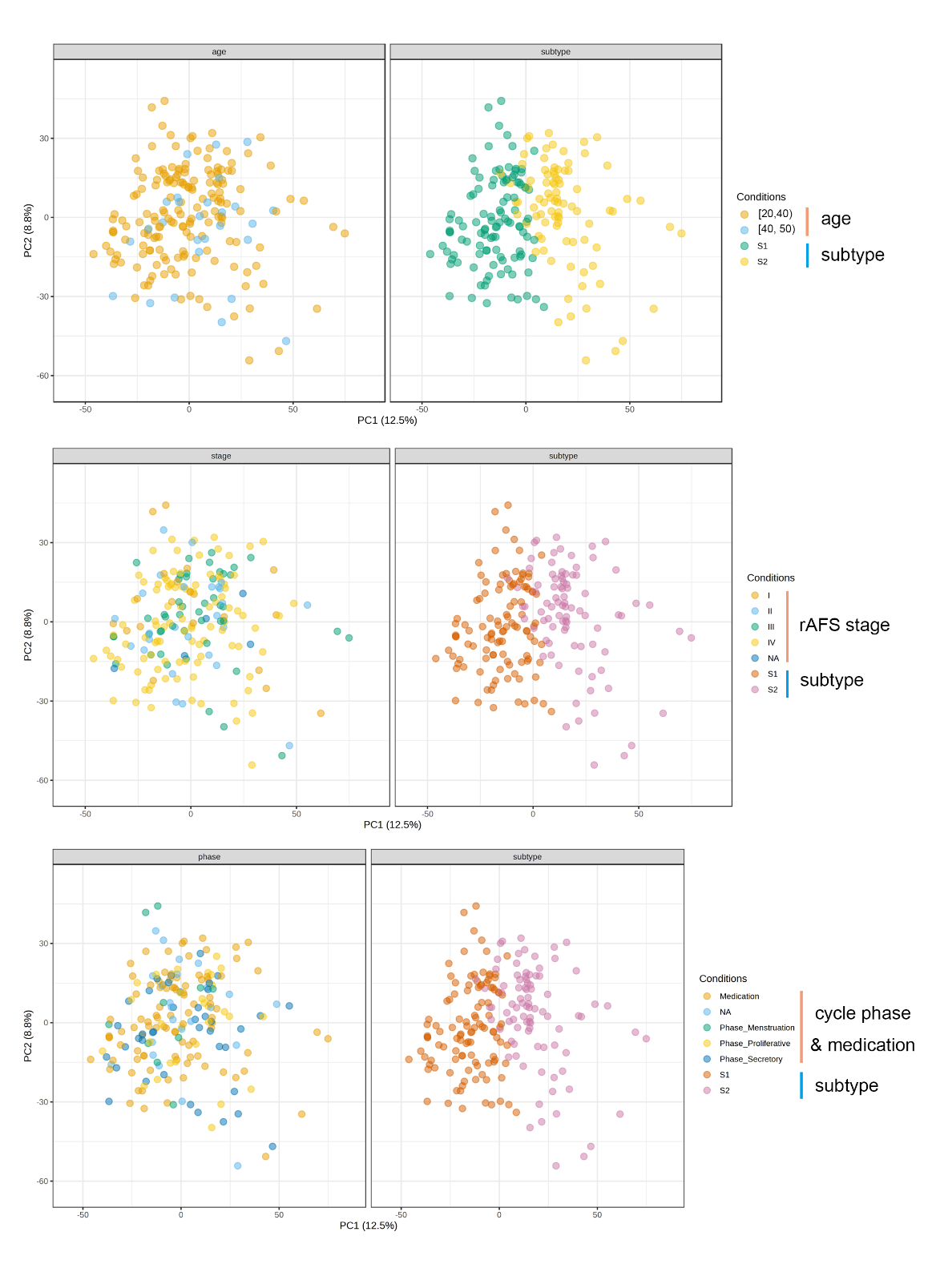


Supplementary Figure 2. The principal component analysis to explore the influence of clinical information on subtypes of endometriotic lesions. Lesions with different clinical covariates (age, rAFS stage, cycle phase and hormone medication) are evenly distributed between S1 and S2. NA: not available; rAFS Stage: revised American Fertility Society stage.


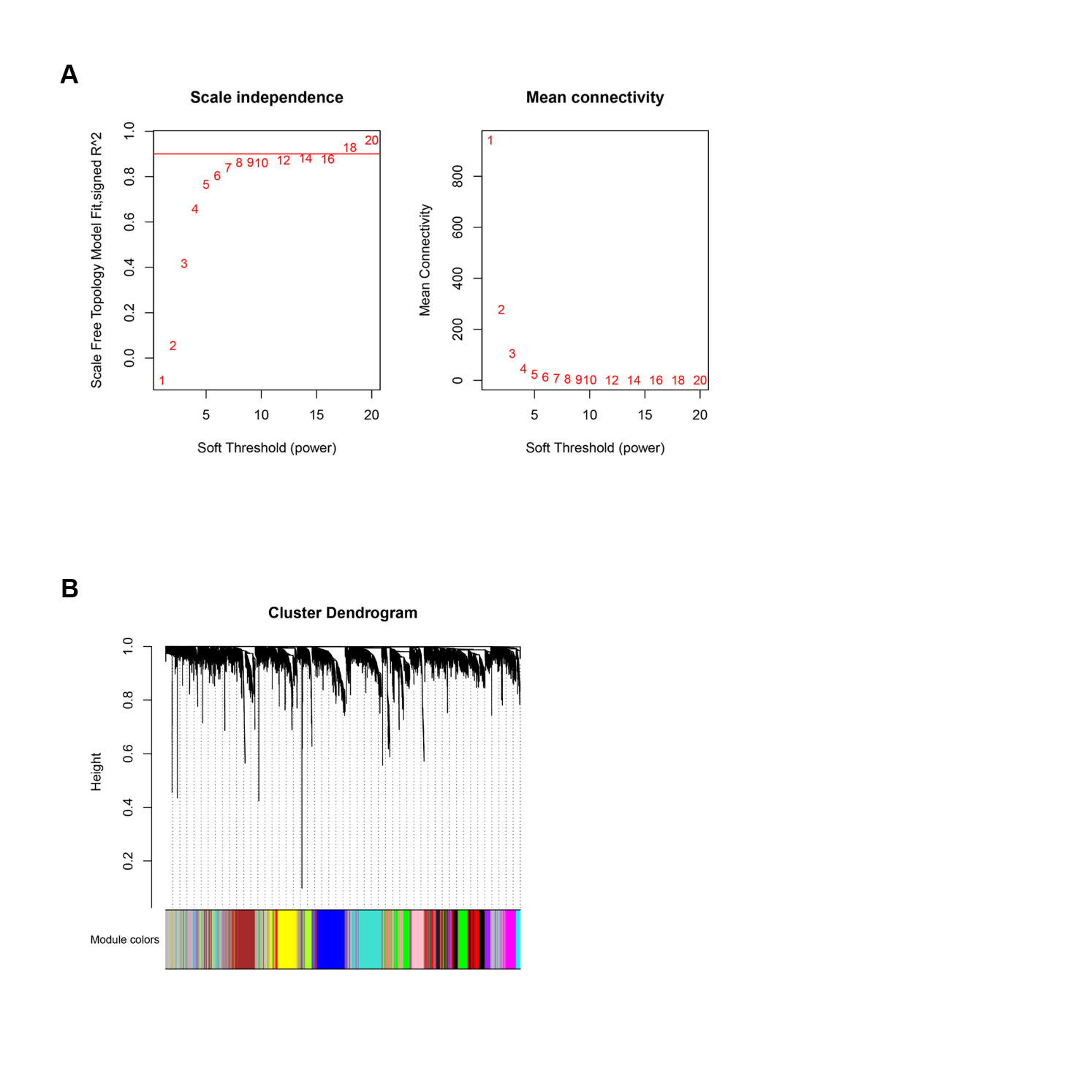


Supplementary Figure 3. (A) Determination of soft-threshold(power) in the WGCNA. (B) Clustering dendrogram of detected co-expression clusters with corresponding color assignments.


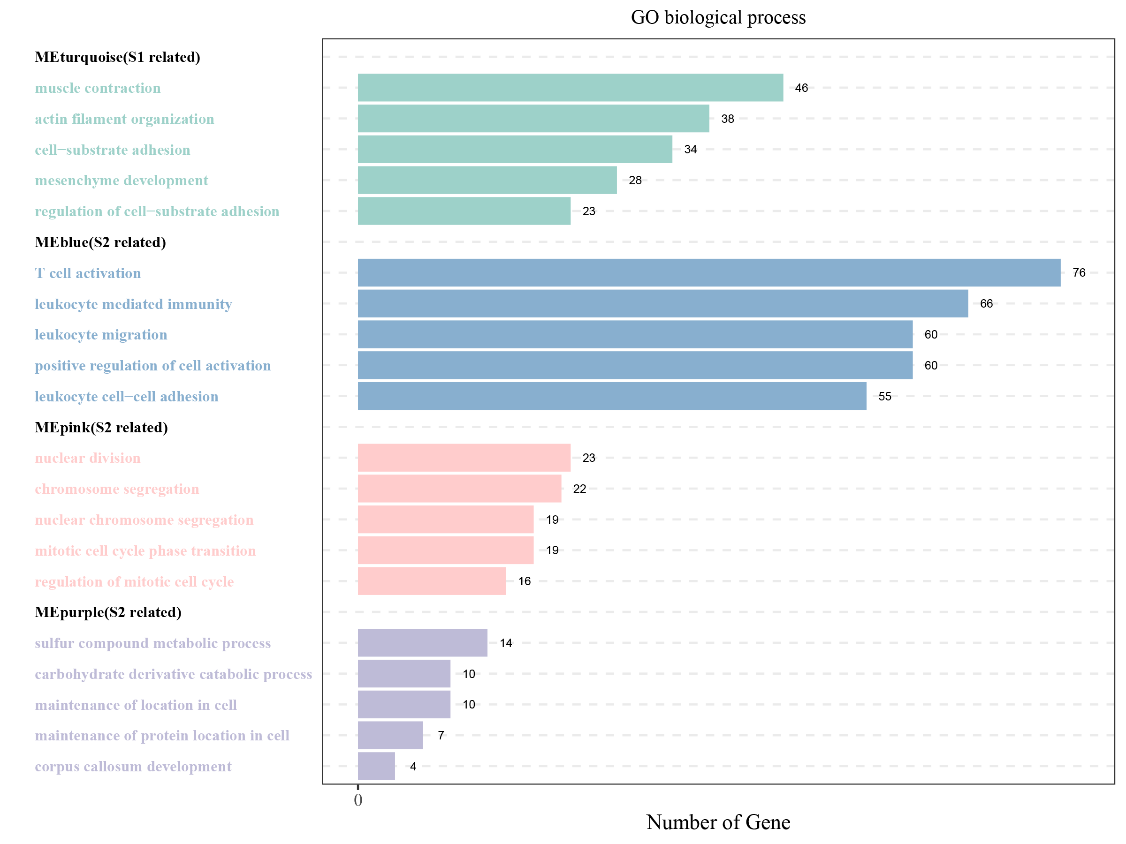


Supplementary Figure 4. Gene Ontology (GO)-biological process analysis in modules related to S1 and S2.


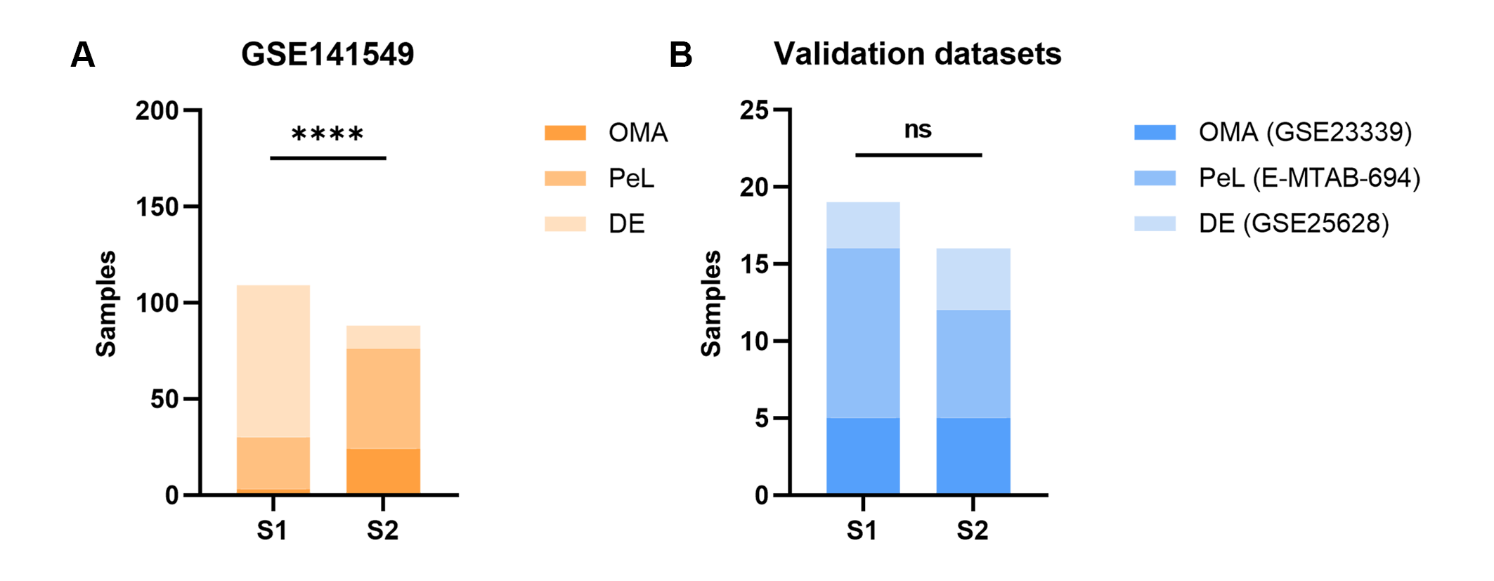


Supplementary Figure 5. The anatomical location of the lesions in the two subtypes. (A) D Distribution of lesion locations in subtypes in the training dataset (GSE141549). (B) Distribution of lesion locations in subtypes in the validation datasets (GSE23339, GSE25628 and E-MTAB-694). OMA: ovarian endometrioma; PeL: peritoneal endometriosis lesions; DE: deep endometriosis; ****: p<0.0001; ns: no significance.

## Supplementary Tables

Supplementary Table 1 Clinical information of 198 lesions in GSE141549 dataset.

| Characteristics | Overall(n=197) | S1(n=109) | S2(n=88) | p |
| --- | --- | --- | --- | --- |
| **Age^a^** | 31.00 [26.00, 37.00] | 31.00 [26.00, 36.00] | 31.00 [25.75, 38.00] | 0.688 |
| **Cycle phase (%)** | | | | **0.788** |
| Menstruation | 10 (5.1) | 8 (7.3) | 2 (2.3) |  |
| Proliferative | 29 (14.7) | 12 (11.0) | 17 (19.3) |  |
| Secretory | 40 (20.3) | 18 (16.5) | 22 (25.0) |  |
| Medication | 96 (48.7) | 60 (55.0) | 36 (40.9) | 0.141 |
| NA | 22 (11.2) | 11 (10.1) | 11 (12.5) |  |
| **rAFS Stage (%)** | | | | **0.068** |
| I | 20 (10.2) | 13 (11.9) | 7 (8.0) |  |
| II | 26 (13.2) | 14 (12.8) | 12 (13.6) |  |
| III | 47 (23.9) | 23 (21.1) | 24 (27.3) |  |
| IV | 100 (50.8) | 57 (52.3) | 43 (48.9) |  |
| NA | 4 (2.0) | 2 (1.8) | 2 (2.3) |  |
| **Lesions (%)** | | | | **<0.001** |
| OMA | 27 (13.7) | 3 (2.8) | 24 (27.3) |  |
| PeL | 79 (40.1) | 27 (24.8) | 52 (59.1) |  |
| DE | 91 (46.2) | 79 (72.5) | 12 (13.6) |  |

a: median [IQR]; NA: not available; OMA: ovarian endometrioma; Pel: peritoneal endometriosis lesions; DE: deep endometriosis; rAFS Stage: revised American Fertility Society stage.
